# Development of Population-Based Cancer Indicators and a Measurement of Cancer Care Continuum Using a Modified Delphi Method

**DOI:** 10.3390/cancers13194826

**Published:** 2021-09-27

**Authors:** Vasuki Rajaguru, Jieun Jang, JaeHyun Kim, JeoungA Kwon, Oyeon Cho, SeungYeun Chung, MiSun Ahn, JinHee Park, YoungJoo Won, KyuWon Jung, Jaeyong Shin, Mison Chun

**Affiliations:** 1 Graduate School of Public Health, College of Medicine, Yonsei University, Seoul 03722, Korea; vasuki@yuhs.ac (V.R.); jieunjang870@gmail.com (J.J.); 2 Department of Health Administration, Dankook University, Cheonan 31116, Korea; kjh930529@gmail.com; 3 Institute of Health Services Research, Yonsei University, Seoul 03722, Korea; kwon.jeounga@gmail.com; 4 Department of Radiation Oncology, School of Medicine, Ajou University, Suwon 16499, Korea; oyeoncho@ajou.ac.kr (O.C.); chungsy@aumc.ac.kr (S.C.); 5 Department of Hematology Oncology, Ajou University School of Medicine, Suwon 16499, Korea; msahn99@aumc.ac.kr; 6 College of Nursing, Research Institute of Nursing Science, Ajou University, Suwon 16499, Korea; jhee@ajou.ac.kr; 7 Division of Cancer Registration and Surveillance, National Cancer Center, Goyang 10408, Korea; astra67@ncc.re.kr; 8 National Cancer Control Institute, National Cancer Center, Goyang 10408, Korea; ara@ncc.re.kr; 9 Department of Preventive Medicine, College of Medicine, Yonsei University, Seoul 03722, Korea

**Keywords:** cancer, Delphi, measurement, cancer indicators, population, monitoring

## Abstract

**Simple Summary:**

This study intended to identify the current state of national cancer management and prevention policies and provide a useful framework for policy establishment. The development of population-based cancer indicators project was conducted as part of a Nationwide Population-based Cancer Registration Project and aimed to applying quality of health care system approaches to the cancer care in population-based monitoring system. The literature review and grey literature review were conducted prior to Delphi method. Our findings confirm 26 cancer indicators and classify them into “Primary prevention,” “Secondary prevention,” “Treatment,” “Quality of care,” “Survivor management,” and “End-of-life care.” The Donabedian model used for health services and the Institute of Medicine quality of healthcare domains of six dimensions were applied to the measurement system. The developed cancer indicators and measurements will be able to provide useful information for prioritizing the operational tasks that can be used by health authorities and policymakers from the other countries.

**Abstract:**

To identify population-based cancer indicators and construct monitoring systems for the entire lifecycle of cancer patients using a modified Delphi method. A modified Delphi method was used to identify the cancer indicators and measurement by scoping review and gray literature. The final list of cancer indicators was developed by consensus of 11 multidisciplinary experts over multiple rounds and rating scored the importance of each indicator on a 10-point scale. Frequency analysis was performed to rate with median scores ≥7 and finalized the list of indicators according to the priority. Initially, 254 indicators were identified, of which 94 were considered important and feasible. After two rounds of rating by the experts and panel discussions, 26 indicators were finalized in six domains: primary prevention (*n* = 7), secondary prevention (*n* = 11), treatment (*n* = 2), quality of life (*n* = 4), survivor management (*n* = 1), and end-of-life care (*n* = 1). The Donabedian model used for examining health services and the Institute of Medicine quality of healthcare domains were applied to the measurement system. Panel experts identified cancer indicators based on priorities with a high level of consensus, providing a scrupulous foundation for community-based monitoring of cancer patients.

## 1. Introduction

The global cancer burden has risen to 18.1 million cases, and 9.6 million cancer deaths occurred in 2018 [1]. Cancer incidence and mortality are rapidly increasing worldwide. During 2012–2016, people diagnosed with cancer were 69% as likely to survive for at least 5 years after being diagnosed as was the overall population [2].

In Korea, one in four deaths per year is attributable to cancer, and more than 200,000 new cancer cases were diagnosed in 2014 [3]. The mid-year population and cancer mortality data from 1983 to 2016 were obtained from Statistics Korea [4]. The number of cancer incidences and deaths are expected to increase with the increase in aging population and adoption of a westernized lifestyle [5].

In this context, cancer statistics are the most important indicators of the national cancer burden and can be used to develop cancer prevention and control strategies [6]. To date, most of these efforts have focused on measuring regional performance [7]. Healthcare cancer indicators, as well as performance indicators or quality measures, are used worldwide to quantify, grade, monitor, and improve the quality of healthcare [8]. The identification and management of care for patients with cancer are important but complex [9]. In addition, cancer has a very significant effect on the patient’s physical, psychological, and social well-being, and various medical professionals are involved in the prevention, diagnosis, treatment, and follow-up care of these patients. Therefore, complexity can lead to an imbalance in care, resulting in discontinuity of cancer care [9,10].

To date, development of indicators related to cancer management, have focused one of the following, national cancer screenings or early detection [11,12], diagnosis [13,14], treatment [14], quality of care [15,16,17,18], survivor management [19,20], and end-of-life care [21]. Although there has been much work involved in the development of indicators related to the quality or performance of specific types of cancer [16,17,18,19,20,21], minimal attention has been paid to the lifecycle of cancer or cancer care continuum.

Our previous preliminary study described the systematic development of cancer indicators based on the entire lifecycle of cancer care as the first step in population-based monitoring. In addition, indicators were grouped into prevention, treatment, quality of care, survivor management, and end-of-life care domains. The participants of measurement system were patients, healthcare providers, and administrators or managers with results based on the prioritized cancer indicators and supporting evidence. We chose a modified Delphi approach to develop cancer indicators and measurements for this project [22]. The Delphi methodology is commonly used when sufficient evidence is available to derive indicators, which require review and deliberation by experts [23]. This study aimed to develop the indicators that were selected by expert consensus. In addition, panel discussion and the Delphi method were used to finalize the prioritized cancer indicators and population-based measurement system as a community-based measurement system.

## 2. Materials and Methods

This study was performed using a well-structured step-by-step approach. The approaches were (1) scoping review, (2) gray literature based on national and international guidelines of cancer care, and (3) expert opinions and panel discussions. Expert opinions were obtained through email, and the panel discussion was conducted with face-to face meeting and online discussion facility was arranged for the experts who could not physically attend discussion due to the coronavirus disease (COVID-19) pandemic.

### 2.1. Literature Search

A scoping review was conducted to develop the first draft of a list of cancer indicators and followed the six stages outlined in the Joanna Briggs Institute manual [JBI, 2015-18] within the compliance of Preferred Reporting Items for Systematic Reviews and Meta-Analyses (PRISMA) statement for scoping reviews [24]. PubMed, CINAHL, Cochrane library, Ovid-EMBASE, RISS, KISS, and KoreaMed databases were systematically searched from May 2020 to September 2020 using a predefined search strategy. Article selection and data extraction were independently performed by two researchers. Articles were included if they defined, described, or recommended appropriate cancer care.

### 2.2. Gray Literature

At the time of the review of literature report submission, our expert committee was instructed to identify additional published and unpublished (government reports, policy statements and papers) references based on cancer care guideline. Therefore, this study diverted to conduct gray literature additionally. Gray literature is composed of knowledge artifact review process, it has been more specifically conceptualized in narrow and broad ways [25]. In extension, we have searched an existing national and international guideline for the entire cycle of cancer care from the official site of the American Society of Clinical Oncology and European Union guidelines, Guidelines of the World Health Organization, International Agency for Research on Cancer, Organization for Economic Cooperation and Development, National Health Service. Guidelines from Canada, Japan, and South Korea were followed, and certain non-English guidelines were translated by the research team. Furthermore, problems resulting from unclear translation or unclear formulation were resolved by discussion. The sources of grey literature and characteristics are given in Supplementary Table S1.

The next process was initiated by combining both the literature review and information acquired from the guidelines. Each concept was converted into an indicator by formulating a definition, numerator, and denominator. All converted topics were checked for loss of information due to translation by a research team.

### 2.3. Delphi Rounds

This study adapted the RAND-modified Delphi panel process, validated by the University of California Los Angeles, which involves a 2-round process of multidisciplinary experts, combining scientific evidence and their experiences to rate the proposed measures [26].

A multidisciplinary panel included forty-two experts to select the cancer measures according to the priority. We divided the experts into three groups: researchers, policymakers, and clinicians. Then, we randomly selected five researchers, three policymakers, and three clinicians. Of the five researchers, two were from internal research group, and the other three from the national cancer center and other colleges. For the three policymakers, one was a director of a public or community health service center, another from the Ministry of Health and Welfare, and the other from the National Cancer Center. For clinicians, we selected one oncologist, one internal physician, and a radio-oncologist. All panel members were contacted via email; the purpose of the project was explained, and consent was obtained.

The literature search yielded 254 indicators, which were reassessed by three rounds of the Delphi method to finalize the appropriate cancer indicators as follows: (1) First round conducted a modified Delphi method, which involved rating or scoring by experts (using frequency and mean scores); (2) second round of qualitative suggestions given by experts during the panel discussions (using comments from both the Delphi rounds) were reviewed; (3) sorting and correcting the appropriate indicators based on the priority and possibility of measuring based on the population were performed.

#### 2.3.1. Round 1

A list of potential indicators was prepared using an Excel spreadsheet based on the literature search and sent to all the experts via email along with a list of references from which the potential indicators were extracted. The experts were asked to rate each indicator based on a 10-point Likert scale by the following criteria: “highly important, direct implementation, long-term monitoring care”. The experts were asked to express their opinions and comment on each indicator.

All the opinions of the experts were collected and entered in a Microsoft Excel database, the mean and frequencies were calculated, and a summary report was prepared. The indicators were organized based on whether a strong consensus for acceptance was achieved with a mean score of ≥8 obtained from the panel members. Information on the selected indicators was distributed to the panel members as the basis for discussion at the in-person meeting.

The first-round analysis revealed a set of 94 indicators that were recommended to be ensured by the experts. All potential indicators were reviewed and confirmed based on the discussion among the research team members who either accepted or rejected these indicators. The research team considered the expert comments and the indicators proposed in the internal meeting. This discussion allowed for the outright rejection of some indicators and the merging of several indicators. The panel experts were asked to rate the indicators in a manner like that of Round 1. Responses were analyzed again; a mixed online and offline mode of discussion was conducted to confirm the acceptance or rejection.

#### 2.3.2. Round 2

The cancer indicators remaining after Rounds 1 were subjected to another panel discussion as Round 2. All panel experts could not be present in person due to the COVID-19 pandemic; therefore, a mixed mode using offline and online Zoom discussions was performed, to facilitate and encourage interaction to share their opinions. The criterion for strong consensus for acceptance or rejection was based on whether a majority of responses was obtained in favor of or against an indicator, respectively.

### 2.4. Quality of the Healthcare Measurement Framework

**Figure 1:**
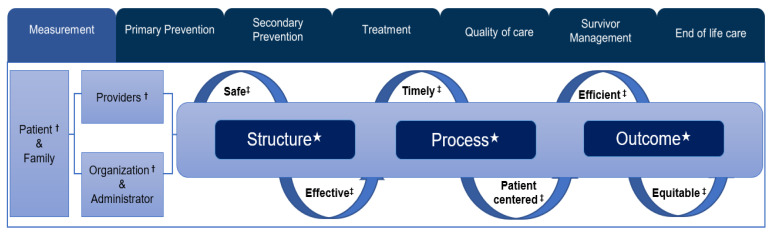
Overview of classification and monitoring unit of the entire life cycle of cancer. † Decision-making authority; ‡ Institute of Medicine’s (IOM) six dimensions of health care quality; ★ Donabedian’s components of quality of care.

In this study, the framework for the quality of healthcare measurement focused on three models: (1) the decision-making authority, (2) Donabedian’s quality of healthcare, and (3) Institute of Medicine’s medical quality care (Figure 1).

The framework for monitoring the measurement of cancer indicators is shown in Figure 1, which presents the concept of the decision-making process involving the patient and their family, healthcare providers, and administrators from organizations. The Donabedian model [27] evaluates three concepts of quality of care that underpin improvement of the care provided. The three components are structure, process, and outcomes. The six domains of healthcare quality [28] are interrelated with both the decision-making authority and healthcare quality measurements.

## 3. Results

### 3.1. Literature Search and Gray Literature

The scoping review search retrieved a total of 6202 articles from electronic databases, and 3234 were selected after eliminating duplicates. There were 310 articles screened for eligibility after carefully reading the abstract and title. Total of 275 articles that did not meet the selection criteria were excluded after careful revision; and the remaining 35 articles were included in the final analysis.

The search of gray literature revealed more than 600 indicators based on the different types of cancer, diagnosis, treatment, quality of care, survivor management, and end-of-life care. All research teams are involved in the analysis and synthesis of the national and international guidelines. We have finalized 254 indicators, and extraction was performed based on the importance and nationwide demand for the indicators (Supplementary Table S1).

### 3.2. Selection Process of Cancer Indicators in the Delphi Rounds

**Figure 2:**
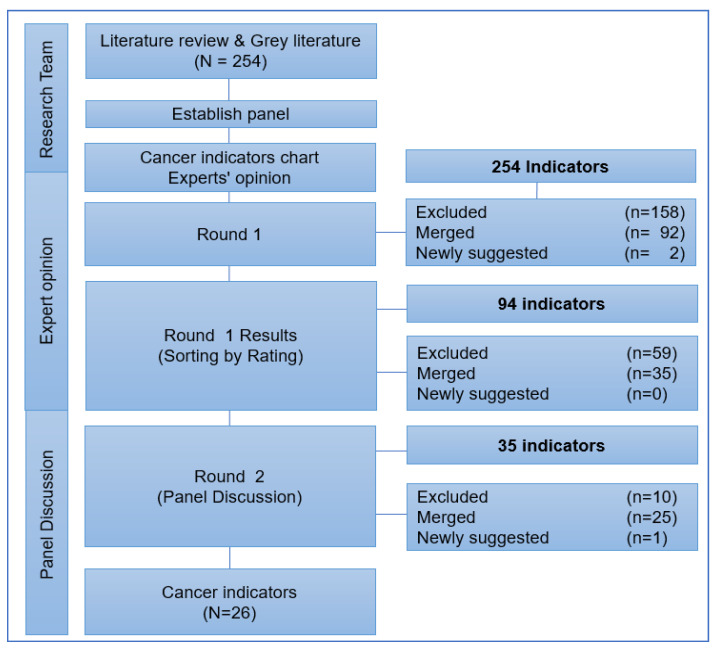
Development of cancer indicators; flow diagram illustrating the development of cancer indicators by expert opinion and panel discussion.

The selection process of cancer indicators is shown in Figure 2. After several meetings and discussions by the entire research team, 254 unique indicators were selected for the panel discussions.

In Round 1, there were 160 indicators excluded according to experts’ opinion based on the importance and need for these indicators. A total of 94 indicators were selected for the next level of discussion. After Round 1, exclusion of indicators was performed by the research team based on the experts’ opinions and the priority of the indicators and 59 indicators were excluded. Total of 35 indicators were finalized for the next round of panel discussion. A total of 26 indicators were selected by the panelists via discussions in Round 2. The characteristics of the selected indicators are listed in Table 1.

### 3.3. Distribution of Cancer-Related Monitoring Indicators According to Donabedian’s Quality of Healthcare

The selected 26 indicators are analyzed by using Donabedian’s quality of healthcare measures for quality of care throughout the lifecycle of cancer. The results shown in Table 2 indicate that most of the indicators were related to the outcome (20, 76.9%), process (4, 15.4%), and structure (2, 7.7%) domains, with maximum involvement of the administrator (14, 53.8%), followed by a combination of the patient and administrator (7, 26.9%), whereas the involvement of provider was lesser (2, 7.7%) than that of others.

**Figure 3:**
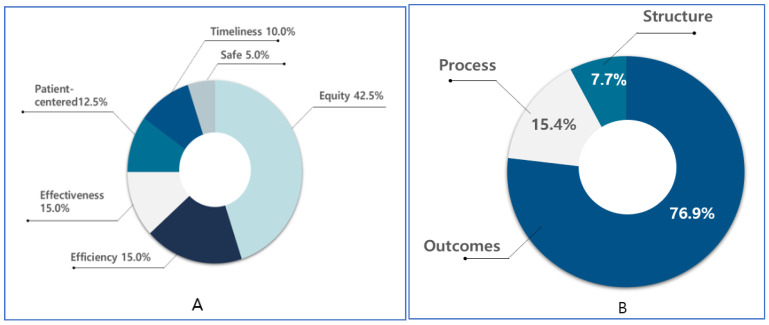
Results of the cancer indicators according to the parentheses of (**A**). Institute of Medicine (IOM) six dimensions of quality improvement. (**B**). Donabedian’s conceptual framework for evaluating health care quality.

According to the classifications, most of the indicators focused on secondary prevention (11, 42.3%) and primary prevention (7, 26.9%), followed by the quality of care (4, 15.4%), treatment (2, 7.7%), and palliative care (1, 3.8%) (Figure 3).

## 4. Discussion

Quality of care initiatives have become common among cancer care providers, and policymakers strive to systematically measure and improve the quality of cancer care. In the present Delphi method, we defined criteria for including and excluding ratings in the in three rounds [12]. For the first Delphi round, we used the median scores given by the experts for each indicator for inclusion or exclusion as the median is less affected by outliers, which are important in group discussions. We had a strict exclusion criterion (median score ≤ 5 on a scale of 1–10) and inclusion criterion (median score ≥ 7 on a scale of 1–10) in the first round of Delphi; therefore, only indicators with a strong agreement among the expert’s opinions are included.

Although the cancer indicators were chosen from or among existing measures in the literature, the perspective and framework used for selection are unique as most quality measurement efforts to date both within and outside cancer have been disease-focused or procedure-focused [12,13,14,16]. Incorporating this perspective into the development of measurement systems based on cancer care indicators for the population is very important to minimize the risk that quality improvement initiatives pose in widening the gaps in the quality of cancer care [8].

The most unique aspect of our study is the perspective method by which the indicators were selected. Cancer is a leading cause of morbidity and mortality in Korea. Furthermore, the diagnosis and treatment of cancer, even if the outcome is not fatal, has a significant impact on the quality of life, morbidity, and healthcare utilization. More importantly, the available literature points to significant differences in cancer screening and treatment with respect to factors such as socioeconomic status [9], health behavior [9,10,13], screening, and treatment process [15,16,17,18], palliative care, and end-of-life care [14,17,19,20,21], highlighting the need to incorporate these perspectives into cancer measurement for the total population.

Another unique aspect of our study is the comprehensive selection of cancer indicators and measurement strategies. Our indicators are valid for the entire lifecycle of cancer from screening through end-of-life care and across the different types of cancers, all of which are important causes of morbidity and mortality in the total population. Specifically, the selected indicators will allow us to look at differences in cancer care in different aspects of the healthcare delivery, including primary care (cancer screening), secondary care (early diagnosis and interventions), tertiary care (treatment and special procedures), quality of life care (follow-up care), survivor management (education and regular health visits), and end-of-life care (palliative and hospice care). This will allow us to determine how different sociodemographic factors influence the quality of cancer care in different healthcare sectors.

Furthermore, we have identified a number of indicators that can be used in a population based on structure, process, and outcome of cancer indicators according to the Donabedian’s model [27]. Studies have concluded that a comparison of outcomes is challenging at the national and international levels because of the non-parametric nature of the indicators [11,12,20,22,23,24]. Our assessment of indicators showed that the structure of cancer care is not a high priority in the healthcare system of Korea, which is already well established. Regarding the process, we could identify six indicators in secondary prevention and survivor management. Most of our indicators were outcome-focused, which is very important for the quality of healthcare systems.

Post this analysis, we have used these indicators to measure cancer care in the total population and identify several gaps in care that are currently being provided. We hope that our cancer indicators and population-based measurements will be of use directly or indirectly to other countries or regions interested according to their organizational pattern to improve their quality of cancer care.

## 5. Strengths and Limitations

This study had several strengths. First, a step-by-step process of indicator selection, including scoping review, gray literature surveying of existing national and international guidelines, and three rounds of the panel discussions were considered as a very rigorous cancer indicator identification procedure. Second, the panel meeting involved experts from all provinces of Korea, which contributed to an efficient and systematic discussion of all prioritized cancer indicators.

We have certain limitations in this study. First, there were only 11 experts involved in the Delphi rounds due to the COVID-19; optimal size and composition of the panel need to be obtained in the further study. Second, the third consensus round was conducted online and offline due to the social distancing. Therefore, most of the panel members did not have a face-to-face interaction, all of them were contacted by zoom meeting at the time of panel discussion. Third, the selected cancer indicators were not submitted for external review. However, the limited involvement of external reviews can be adequately addressed in future research. Four, further work, which may include a review of the potential baseline indicators such as diet, family history, patient satisfaction, information systems, and patient experience measures to identify relevant data items and develop new data items where required, is necessary to support the implementation of these indicators. There is a need to examine the extent to which the measurement of an indicator may contribute to changes in service provision and patient outcomes and identify the mechanisms by which service delivery processes affect patient outcomes.

## 6. Conclusions

The Delphi method provided guidance in selection of indicators that are widely accepted by those who are willing to improve population-based cancer care. These indicators are highlighting the combination of clinical and community-oriented cancer care practice, health care professionals, communication, and quality of health care system. Currently, there is substantial interest in the development of cancer indicators. Thus, not only the process but also the resulting indicators may prove of interest to policymakers, clinicians, and researchers across South Korea.

## Figures and Tables

**Table 1:** Description of selected cancer indicators.

No.	Domains	Participants	Sub Domains	Indicators	Measurement/Total Population	Results
1	Primary prevention	Patient	Obesity	Obesity rate	- The percentage of people with a body mass index (BMI) of 25 kg/m^2^ or more.	Importance
2			Physical activity	Physical activity/Exercise rate	- The percentage of people who regularly exercised moderately.	Importance
3		Patient/Administrator	Alcohol consumption	High risk of Alcohol consumption rate	- The percentage of people diagnosed with hypertension or diabetes compared to the population in the relevant treatment area.	Importance
4			Risk of Chronic disease	Current status of Cancer-related chronic diseases	- The percentage of people diagnosed with hypertension compared to the population in the relevant treatment area.	Importance
5			Smoking	Average daily smoking amount per person	- The average number of cigarettes smoking per day by current smokers.	Importance
6	Secondary prevention	Patient/Administrator	Smoking	Smoking rate	- The percentage of current smokers among those who smoked more than 5 packs (100 cigarettes) in their lifetime.	Importance
7		Administrator	Infection	Helicobacter infection rate	- The percentage of the population infected with *H. pylori* compared to the number of people over the age of 40 years underwent gastroscopy during national cancer screening.	Selective
8		Patient	Incidence	Probability of Developing Cancer (Cumulative incidence rate)	- Survival and life expectancy, cumulative cancer occurrence probability.	Importance
9		Administrator	Incidence	Cancer incidence rate (Crude Rate)	- The number annual occurrences divided by the 100,000 population.	Importance
10			Screening	Cancer Incidence (Age-Standardized Rate)	- The means and weighted average of incidence rate calculated by weighting the proportion of the standard population corresponding to each age group.	Importance
11			Screening	Cancer Screening Rate	- The percentage of eligible people who received cancer screening through the national cancer screening program among the six cancer types included in the national cancer screening program.	Selective
12			Screening	Positive Predictive Value of Eligible Persons in National Cancer Screening Examination	- This indicator measures whether national cancer screening providers (clinicians, hospitals) within a specific clinical privilege have an appropriate level.	Selective
13	Secondary prevention	Administrator	Health professional	Providers’ Excellence in National Cancer Screening Program	- Assess whether national cancer screening providers (clinics, hospitals) within a specific medical field have an appropriate level.	Selective
14			Health professional	Regional relevance index of national cancer screening candidates	- The proportion of the first registered national cancer registration at a medical institution within the Clinical Privilege.	Selective
15			Appropriate procedures	Distribution of invasive extent (stage) at diagnosis	- The percentage of cancer occurrence by summary stage at the time of cancer diagnosis.	Selective
16		Patient/Administrator	Screening	Colonoscopy screening rate of national cancer screening	- The percentage of people who received colonoscopy within 12 months among those who need colonoscopy in national colon cancer screening examinees.	Selective
17		Patient/Administrator	Screening	Cancer detection rate among national cancer screening candidates	- The percentage of cancer patients among national cancer screening examinees. - Index showing the degree of early detection of cancer through national cancer screening.	Selective
18		Patient/Administrator	Prevalence	Cancer prevalence rate (Crude rate)	- The number of sick people in a specific population divided by the total population during the observation period, generally expressed as a rate per 100,000 population.	Selective
19	Treatment	Health care provider	Health system	Mean Sojourn Time between National Cancer Screening and Confirmative Diagnosis	- The period of time (days) from the national cancer screening to the date of registration of cancer, defined as a proxy indicator showing the effectiveness of the cancer-related medical delivery system. - Calculated for the group who received a diagnosis of cancer within 12 months after finding a national cancer screening.	Selective
20		Health care provider	Appropriate treatment	Immuno-histopathologic Test for Breast Cancer Patients	- The indicator defined whether breast cancer immunohistochemistry was performed to provide appropriate treatment to breast cancer patients.	Importance
21	Quality of care	Administrator	Survival/Mortality	Cancer mortality rate	- The indicator measures the annual number of deaths due to cancer divided by the annual population is calculated to be 100,000 secretion.	Importance
22		Administrator	Survival/Mortality	5-year relative survival rate	- The indicator measures the observed survival rate of patients with the disease of interest (carcinoma), divided by the expected survival rate of the general population and age group.	Importance
23	Quality of care	Administrator	Survival/Mortality	5-year absolute survival rate	- The indicator measures the probability that a cancer patient will be alive for a certain period (5 years, 10 years) is intended to calculate the survival rate without considering the cause of death.	Selective
24			Survival/Mortality	Disability-adjusted Life Year (DALY)	- The indicator calculates the sum of the number of years of survival lost due to premature death and the number of years of health lost due to disability due to disease.	Selective
25	Survivor management	Administrator	Health screening	National Health Screening Rate of Cancer Survivors	- The indicator measures the percentage of examinees who underwent general health examination through the national general health examination program among those subject to national general health examination.	Selective
26	End-of-life care	Administrator	Health care professional	The availibility of beds at Palliative Care Institutions	- The indicator calculates the number of beds operated in hospice palliative care institutions.	Selective

**Table 2:** Distribution of selected cancer indicators according to quality of health care measurement based on (A). Donabedian’s model for evaluating health care quality and (B) Institute of Medicine (IOM) six dimensions of quality improvement.

Measurement	Subjects/Participants	Primary Prevention	Secondary Prevention	Treatment	Quality of Care	Survivor Management	End-of-Life Care	Total N (%)
A	Patient	2	1					3 (11.5)
	Providers			2				2 (7.7)
	Administrator	1	7		4	1	1	14 (53.8)
	Patient/ administrator	4	3					7 (26.9)
	Total N (%)	7 (26.9)	11 (42.3)	2 (7.7)	4 (15.4)	1 (3.8)	1 (3.8)	26 (100)
B	Safety		2					2 (5)
	Effective		1		4	1		6 (15)
	Patient-centered		2	2			1	5 (12.5)
	Timely		3	1				4 (10)
	Efficiency		4		1		1	6 (15)
	Equity	7	7	1	1		1	17 (42.5)
	Total N (%)	7 (17.5)	19 (47.5)	4 (10)	6 (15)	1 (2.5)	3 (7.5)	40 (100)

## Data Availability

The data presented in this study are available on request from the corresponding author.

## Supplementary Materials

The following are available online at https://www.mdpi.com/article/10.3390/cancers13194826/s1, Table S1: Characteristics of the National and International Cancer guidelines
