# Supplementary material for: Development of Population-Based Cancer Indicators and a Measurement of Cancer Care Continuum Using a Modified Delphi Method
Source: Cancers (Basel). 2021 Sep 27;13(19):4826. doi: 10.3390/cancers13194826 (PMC8508364; doi:10.3390/cancers13194826)
Supplement: Supplementary file 1 [file cancers-13-04826-s001.zip › cancers-1388523-SI.pdf]

Supplementary Table S1. Characteristics of the National and International Cancer guidelines

Level	Cancer Guidelines and Reports	Organization	Publication	Population Coverage	No.of Indicators
International Guidelines	Cancer control and world cancer report (46)	International Agency for Research on Cancer (IARC)	2009-2019	International	127
	Canadian cancer society (47)	CANADA	2015-2019	National, Regional	122
	European guide for quality national cancer control programs (48)	Europe	2013-2019	National	101
	National cancer care plan (49)	The Organization for Economic Co-operation and Development (OECD)	2013-2019	International	150
	Quality oncology practice initiative (50)	American Society of Clinical Oncology (ASCO)	2013-2019	National	98
	Scottish cancer task force national cancer quality steering group guidelines and indicators (51)	National Health Service (NHS)	2013-2020	National	116
	WHO-Cancer care Report for ALL (52	World Health Organization (WHO)	2005-2020	International	268
	National cancer registration hospitals such as cancer treatment-linked hospitals (53)	Center for Cancer Control and Information Services, National Cancer Center- Japan	2017	National	12
	Cancer hospital- five-year survival rate report 2010-2011 (54)		2019	National	12
	Cancer care linked hospital- Survival rate report – 2013 (55)		2019	Regional	7
	Cancer statistics 2019 (56)		2019	National	25
	National screening programme DATA book (57)		2020	National	7
	Monitoring of cancer incidence in Japan (58)		2019	National	15
	Cancer incidence rate in Japan (59)	Cancer and Disease Control Division, Ministry of Health, Labor and Welfare-Japan	2020	National	4
National Guidelines	Cancer statistics (60)	National Cancer Center (NCC) Korea	2007-2020	National	9
	Measures to improve the classification system and evaluation of health indicators (61)	Health Insurance Review and Assessment Service Korea	2019	Regional	1
	Regional health index (62)	Korea Health Ranking (Elio & company)	2018	Regional	1
	Regional health vulnerable indicators (63)	Korea Health Promotion Institute	2016	Regional	8
	Community health Survey – Health indicators (64)	Hallym University	2013	Regional	5
	Community health Survey (65)	Center for Disease Control Korea	2008-2019	Regional	28
